# Early prenatal diagnosis of conjoined twins at 7 weeks and 6 days' gestation with two-dimensional Doppler ultrasound: a case report

**DOI:** 10.4076/1757-1626-2-8330

**Published:** 2009-07-22

**Authors:** M Zeki Taner, Mertihan Kurdoglu, Cagatay Taskiran, Zehra Kurdoglu, Ozdemir Himmetoglu, Sevim Balci

**Affiliations:** 1 Department of Obstetrics and Gynecology, Gazi University, Medical College and HospitalAnkaraTurkey; 2 Department of Obstetrics and Gynecology, Yuzuncu Yil University, Medical College and HospitalVanTurkey (Formerly, Department of Obstetrics and Gynecology, Gazi University, Medical College and HospitalAnkaraTurkey); 3 Van Maternity and Children’s HospitalVanTurkey (Formerly, Department of Obstetrics and Gynecology, Gazi University, Medical College and HospitalAnkaraTurkey); 4 Department of Pediatrics, Division of Clinical Genetics, Hacettepe University, Faculty of Medicine and Ihsan Dogramaci Children’s HospitalAnkaraTurkey

## Abstract

This case report presents the prenatal diagnosis of conjoined twins at 7 weeks and 6 days’ gestation according to the last menstrual period and 6 weeks and 4 days’ gestation according to crown-rump length in a 32-year-old Turkish woman, using two-dimensional Doppler ultrasound. The twins were fused to each other at the thoracic region (thoracopagus). In the light of previous reports of conjoined twins this appears to be one of the earliest prenatally diagnosed cases in the medical literature.

## Introduction

In the last decade, with the advances in assisted reproductive technologies, the number of multifetal pregnancies of high fetal number has tended to rise. Chorionicity and amnionicity are important in the assessment and management of multifetal pregnancies since monochorionic pregnancies have an increased risk of fetal malformations, twin-twin transfusions, morbidity, and mortality [1,2]. The type of monozygotic pregnancies associated with the most severe complications is conjoined twins. Although the prognosis of conjoined twins varies according to the degree and location of conjunction, it is associated with a high perinatal mortality rate. Survivors need surgical correction via a series of major operations and a life with many coexisting diseases is inevitable [3].

In this report, a case of conjoined twins diagnosed prenatally at 7 weeks and 6 days’ gestation is presented.

## Case presentation

**Figure 1.**
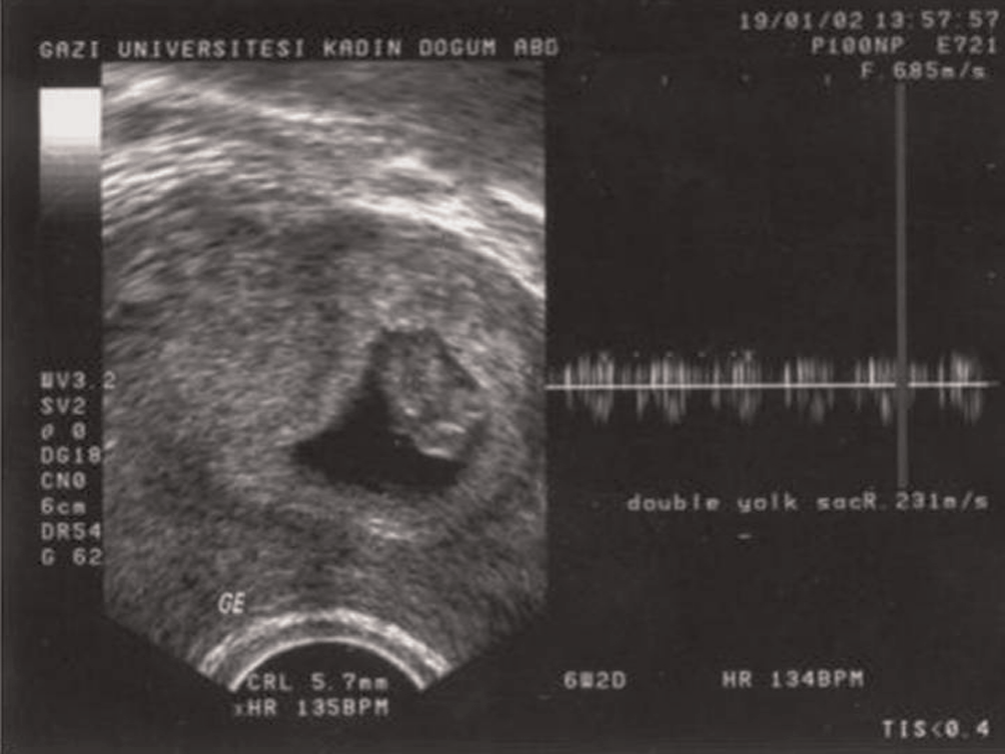
A wide fetal pole with bifid appearance and two yolk sacs in a unique gestational sac raise suspicion about the presence of conjoined twins with different fetal cardiac activities.

**Figure 2.**
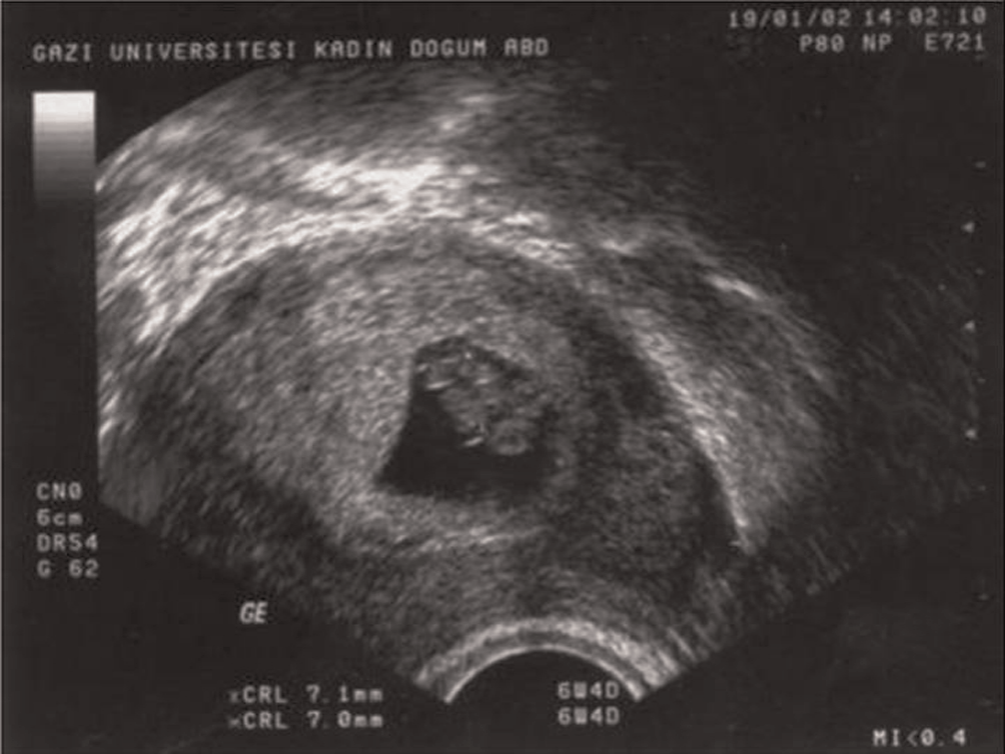
CRL (crown-rump length) measurements of conjoined twins.

A 32-year-old Turkish woman with gravida 3, parity 0, abortus 2, presented with a 7 weeks and 6 days pregnancy according to her last menstrual date. On her first visit transvaginal ultrasonography revealed a wide fetal pole and two yolk sacs in a unique gestational sac (Figure 1). Ultrasonography was performed using a 7.5 MHz transvaginal probe with two-dimensional ultrasonography (Logic 500, GE Medical Systems) in Gazi University Hospital, Department of Obstetrics and Gynecology, Division of Perinatology. Zooming in on the image showed fetal cardiac activity in two different sites on a wide embryonic structure. Heart rate determination with Doppler revealed different fetal cardiac activities of 140/min and 135/min (Figure 1). CRL (crown-rump length) measurements were 7.1 mm and 7 mm, respectively, consistent with 6 weeks and 4 days of gestation (Figure 2). In the ultrasonographic examinations 45 minutes and 1 day later, there were no changes in the embryonic appearance or the positions of the embryos relative to each other. The embryos seemed to be fused to each other at the thoracic region (thoracopagus). The family history of the patient was positive for twining; during her first pregnancy at 11 weeks a singleton sac and embryo were evacuated and during her second pregnancy at 9 weeks two sacs and two embryos were evacuated after the diagnosis of missed abortion.

Since the patient had had recurrent misabortions, she wanted to continue with her pregnancy. However, when the ultrasonography was repeated 2 days later, no cardiac activity of the fetuses was observed. With the diagnosis of missed abortion, the products of conception were surgically evacuated and placental tissue samples were obtained. The tissues were then examined and cleaned by blotting on an absorbent tissue pad. Maternal blood clots were removed from the placenta, and 10-20 mg of placental villi was placed into culture medium. A sample was set up for complete karyotype analysis in the cytogenetic laboratory and cultured. The cytogenetic examination of the material revealed a 46 XX normal karyotype. All pregnancies of this patient were spontaneous and the assays for recurrent gestational losses revealed no pathology. The maternal and paternal karyotypes were 46 XX and 46 XY, respectively, and the parents were not consanguineous. Serum folic acid levels of the mother were within normal limits.

## Discussion

The incidence of conjoined twinning is 1 in 50,000 to 1 in 100,000 births in the world [4]. Conjoined twins, being the most extreme form of monozygotic twinning, occur in about 1% of monozygotic twins. It is proposed that the origin of conjoined twins is at the primitive streak stage of the embryonic plate (15-17 days), and results from an error in blastogenesis due to incomplete fission of a single zygote [5].

The prenatal diagnosis may be suspected and confirmed if two fetuses cannot be visualized separately in a single gestational sac. Bifid appearance of the first trimester fetal pole, presence of more than three umbilical cord vessels, persistency of heads at the same level and body plane, and failure of the fetuses to change position relative to each other over time are other sonographic features that assist in making the diagnosis [6]. The diagnosis in our case was based on these criteria, which were valid for the early gestational weeks. Sebire et al. found nuchal translucency (NT) increment in 6 fetuses of four conjoined twins diagnosed. This observation shows that determination of increases in NT in multifetal pregnancies in the first trimester should alert us about the necessity of careful examination of the case with regard to the possibility of conjoined twinning [7]. However, gestational weeks in our case were too early to allow nuchal translucency to be seen.

Experienced operators can detect conjoined twins from as early as the first trimester with careful and detailed scanning using transvaginal ultrasonography. With the use of color Doppler examination, two-dimensional echocardiography, ultrafast magnetic resonance imaging, and 3-dimensional color sonography with 5-7 MHz transabdominal and transvaginal transducers anatomical malformations in conjoined twins can be investigated precisely, facilitating the counseling of parents of conjoined twins [8]. However, caution must be taken with the interpretation of early gestational examinations, as a false positive diagnosis of conjoined twins has been reported [9]. The diagnosis in our case was achieved using two-dimensional ultrasonography with Doppler technology using a 7.5 MHz transvaginal transducer.

Prenatal suspicion of conjoined twins can arise as early as 7 weeks of gestation [10]. When we consider that in our case the diagnosis was made at 7 weeks and 6 days of the pregnancy according to the last menstrual date and 6 weeks and 4 days according to crown-rump length, this case is one of the earliest prenatally diagnosed cases in the medical literature reviewed in PubMed.

## Conclusions

An early diagnosis of conjoined twins is crucial for the further management of pregnancy and appropriate counseling of the family. It must be stressed that absent separating membrane, conjoined body parts, and inseparable bodies or heads despite changes in fetal positions and bifid appearance of the fetal pole are the most valuable ultrasonographic diagnostic criteria in the first trimester.

## List of abbreviations

CRL: Crown-rump length
NT: Nuchal translucency
